# Drosophila as a Model System to Study Cell Signaling in Organ Regeneration

**DOI:** 10.1155/2018/7359267

**Published:** 2018-03-19

**Authors:** Sara Ahmed-de-Prado, Antonio Baonza

**Affiliations:** Centro de Biología Molecular “Severo Ochoa”, CSIC/UAM, 28049 Madrid, Spain

## Abstract

Regeneration is a fascinating phenomenon that allows organisms to replace or repair damaged organs or tissues. This ability occurs to varying extents among metazoans. The rebuilding of the damaged structure depends on regenerative proliferation that must be accompanied by proper cell fate respecification and patterning. These cellular processes are regulated by the action of different signaling pathways that are activated in response to the damage. The imaginal discs of* Drosophila melanogaster* have the ability to regenerate and have been extensively used as a model system to study regeneration. Drosophila provides an opportunity to use powerful genetic tools to address fundamental problems about the genetic mechanisms involved in organ regeneration. Different studies in Drosophila have helped to elucidate the genes and signaling pathways that initiate regeneration, promote regenerative growth, and induce cell fate respecification. Here we review the signaling networks involved in regulating the variety of cellular responses that are required for discs regeneration.

## 1. Introduction

Regeneration is the ability that presents some organisms and allows them to partially or fully replace missing or damaged organs. This capacity is conserved among different phyla and it involves a wide range of processes, from wound healing to the induction of regenerative growth to reconstruct a whole new organ, as in urodele amphibians [1, 2]. Studying regeneration should shed light on the mechanisms that regulate this process, paving the way for their potential therapeutic applications in regenerative medicine.

*Drosophila melanogaster* is a powerful model system to perform genetic analysis, and it has provided much of the information of our understanding about the genetic basis of organ morphogenesis. While the adult organs of Drosophila are incapable of regenerating, the primordia of these structures, known as imaginal discs, can undergo regenerative growth. Imaginal discs are epithelial sac-like structures that develop from the embryonic ectoderm and after a period of cell proliferation in the larval stages, they give rise to the adult cuticle.

A series of classic experiments from the mid-1940s to the 1970s laid the groundwork for our current understanding of regeneration in Drosophila imaginal discs [3–5]. The classic approach used to study regeneration in Drosophila was to grow the regenerating discs* in vivo* culture. The disc was extracted from the larva and after amputating a fragment of it, it was transplanted into the abdomen of an adult host, where the cells of the discs could proliferate and the disc would regenerate [3, 6]. After regeneration the disc was then extracted from the adult host and examined [5, 7].

**Figure 1:**
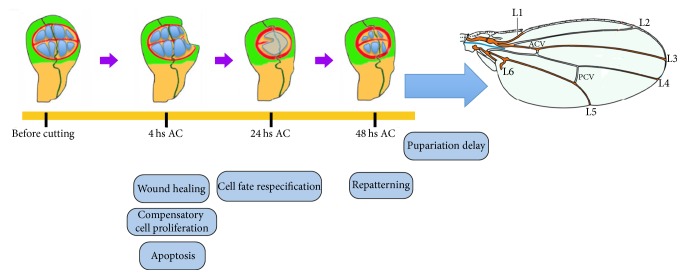
*Drosophila* wing imaginal discs regeneration process. After a cut or genetic ablation in the third instar wing imaginal discs various cellular processes occur. First, four hours after cut (AC), the wound heals restoring the epithelial continuity. Around 24 hours AC, some cells lose expression of markers of cell fate commitment. Finally, the pattern is restored and the discs give rise to a normal pattern and sized adult wings. However, there is a delay in pupariation to allow the tissue to regenerate.

One of the problems of these studies is that regeneration did not occur under physiological conditions, since the disc regenerates in an adult host. Moreover, the process of disc extraction and transplantation will cause some stress to the cells of the discs, provoking apoptosis and halting cell proliferation in the first few hours after transplantation [8]. An alternative approach based on the transient induction of cell death in specific regions of the discs has resolved some of these problems. This method implies the use of the* Gal4/UAS* binary system in combination with* Gal80*^*ts*^ to express a cell-death inducer, which makes it possible to genetically ablate a region of the disc* in vivo *for a predetermined period of time, after which the disc recovers [9, 10]. Although this technique mimics some aspects of surgical amputation, there are important differences between these two approaches. For instance, unlike amputation the overexpression of a cell-death inducer may not be sufficient to eliminate all the cells in the region targeted, and thus dying cells will coexist with living cells during the period of recovery. A further method has been developed to study disc regeneration in its normal developmental context. This system consists of removing a section of the disc “in situ” inside the larvae without extracting the discs from the larvae [11, 12]. The results obtained using these different approaches revealed that the initial stages of disc regeneration involve different processes. Thus, after wound healing a zone with a high rate of cell proliferation appears at the edges of the wound, similar to the blastema that originates during limb regeneration in amphibians and teleost fish. In addition, there is a temporary loss of markers of cell fate commitment and repatterning (Figure 1) [5, 7, 10, 12, 13]. All these cellular processes are triggered and regulated by the action of different signaling pathways. In this article we will focus on the signaling networks that regulate the various cell responses required to control the early stages of imaginal disc regeneration.

## 2. Reactive Oxygen Species (Ros) Are Induced in Response to Disc Damage

One of the first reactions of the imaginal disc in response to damage is the production of reactive oxygen species (Ros). Ros have been increasingly implicated in the physiological regulation of many developmental processes, including the emergence of stem cells, inflammatory cell recruitment, or the differentiation of embryonic cardiomyocytes. Ros act at many distinct levels in biological processes, affecting gene expression, protein translation, and protein-protein interactions [14–17]. Ros are the by-products of aerobic metabolism and they include superoxide (O_2_^−^) and hydrogen peroxide (H_2_O_2_). There are numerous potential sources of Ros within the cells and various organelles within the cell can produce Ros. One important generator of oxidant is a family of NADPH-dependent oxidases (Nox/Duox). These transmembrane proteins regulate the generation of Ros and an increase in their activity is produced in response to different stimuli after damage, such as the liberation of Ca^2+^. Indeed, wounding in Drosophila embryos provokes the binding of Ca^2+^ to Duox and its activation [18].

The specific effects of Ros are largely modulated by the reversible oxidation and reduction of reactive Cys residues, which in turn provokes the reversible modification of enzymatic activity of redox-sensitive targets, such as Tyrosine phosphatases or kinases [16, 17].

**Figure 2:**
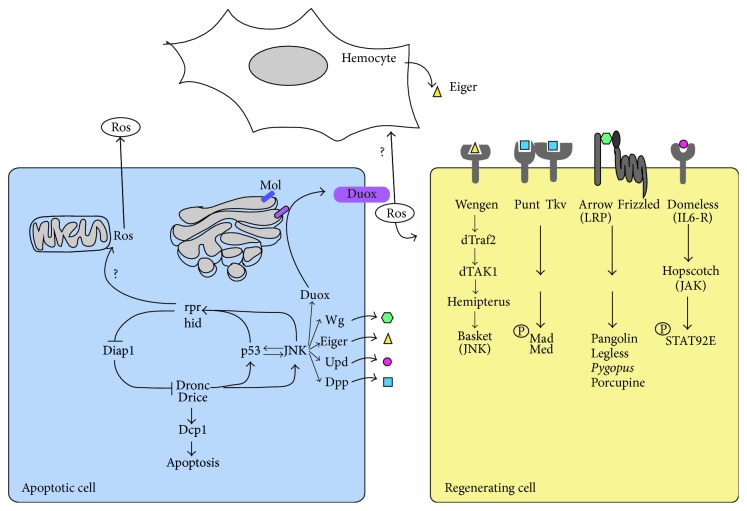
Signaling network promoting apoptosis and Ros production after discs damage. Damage causes the activation of different signals that lead to apoptosis and high levels of Ros accumulation in dying cells. The function of Duox leads to extracellular Ros production that is responsible for hemocyte recruitment and promotes the activation of different signaling pathways in surrounding cells. Hemocytes secrete Eiger, which activates JNK pathway in the adjacent cells. Apoptotic cells produce also different signals that influence surrounding cells.

There is increasing evidence indicating that, during regeneration in vertebrates, the response to damage involves oxidative stress and, consequently, the stimulation of stress-activated protein kinases [19–21]. Ros have been also proposed to play a key role in disc regeneration [22–27]. Physical injury or genetic ablation of part of the imaginal disc provokes Ros production in cells at the wound site [22–25]. The initial burst of Ros acts as a chemoattractant for macrophages and it is necessary for the activation of different signaling pathways [22, 25, 27] (reviewed in [26]) (Figure 2). It is not clear how the first burst of Ros is generated, although different mechanisms have been identified that contribute to the increase in Ros levels in response to damage (see below). Shortly after trauma, Ros is also detected in cells adjacent to the injured region, although at a lower concentration [22].

## 3. Activation of JNK Signaling Promotes Ros Production and Triggers Multiple Responses

JNK signaling is initially triggered at the wound site in response to Ros, and probably by other cellular stress signals [28]. Ros has been proposed to regulate the activity of MAP3K (MEKK1) and apoptosis signal-regulating kinase 1 (ASK1 or MAP3K5), kinases that reside upstream of JNK [16, 17, 29–31]. In addition, Ros can also block the function of the MAP kinase phosphatases that inhibit JNK signaling [30, 32]. JNK signaling promotes the activation of proapoptotic genes like* reaper (rpr), head involution defective (hid), *and* grim.* The proteins encoded by these genes bind to and inhibit the activity of Drosophila IAP-1 (Diap1), which in turn blocks the initiator caspase-9 orthologue Dronc (Drosophila NEDD2-like caspase). Dronc activates the effector caspases Dcp1 and Drice (Drosophila interleukin converting enzyme), inducing apoptosis. It has been shown that* rpr* and* hid* alter cytochrome Cytochrome C in the mitochondria, leading to mitochondrial disruption [33–36]. While the origin of Ros after damage remains unclear, the action of* rpr* and* hid* on mitochondria could favor Ros production by apoptotic cells (Figure 2).

The tumor suppressor Dp53 is another factor activated by JNK signaling and that is necessary to trigger apoptosis, playing a fundamental role in the elimination of cells that cannot complete DNA repair [37]. Both dp53 and JNK can activate each other in a Dronc-independent manner, and they establish a feedback loop that amplifies the initial apoptotic signals [38]. This loop is very important to promote cell death in response to the activation of the apoptotic pathway (Figure 2) [38]. Dp53 is required to induce compensatory proliferation and to reestablish the patterning of the damaged discs. Interestingly, it has been proposed that these functions are not dependent on apoptosis [39, 40].

JNK signaling also increases the levels of Ros by transcriptionally activating the gene* moladietz (mol)* [23]. This gene encodes the Duox-maturation factor NIP that is required for the production of Ros. Therefore, the activation of* mol* favors the production of Ros and it promotes a positive feedback signal that ensures the prolonged JNK activation necessary for regenerative growth (Figure 2) [23, 25]. Ros are also involved in the activation of* Cap-n-collar (cnc)*. The transcriptional targets of* cnc* constrain Ros levels within a range in which regeneration is most efficient [24]. The cells with the highest levels of JNK signaling die and produce high levels of Ros. Indeed, at the wound edge there are apoptotic cells that have high levels of Ros in conjunction with high levels of JNK [22]. Ros propagate from dying or dead cells to the nearby surviving cells, and they activate multiple factors and signaling pathways. Indeed, Ros can propagate from cell to cell through aquaporins [41] or gap junctions [42].

Like other signals generated by apoptotic cells, Ros can affect surrounding cells. Various studies have shown that apoptotic cells can influence the proliferation and survival of nearby surviving cells. Indeed, apoptotic cells have been proposed to send signals that induce surrounding cells to divide or to die; events are known as apoptosis-induced proliferation (AiP) or apoptosis-induced apoptosis (AiA) [43]. It has been suggested that apoptotic cells can release mitogenic factors, such as the Drosophila Wnt1 homologue Wingless (Wg), the bone morphogenetic protein (BMP) Decapentaplegic (Dpp), and the leptin-like (IL-6 family) cytokine ligands Unpaired proteins (Upd, Upd2, Upd3) (Figure 2) (see below). Therefore, apoptosis at the wound site might fulfill a fundamental role in regulating regenerative proliferation. However it has been shown that the partial inhibition of apoptosis does not have a major effect on disc regeneration [8, 44]. A possible explanation for this paradox might be that apoptosis was not fully suppressed in any of these analysis. Thus, the signals emitted by the few apoptotic cells that remain might be sufficient to induce proliferation of the surrounding cells. More studies will be necessary to clarify the true role of apoptotic cells during regeneration.

## 4. P38 and JNK Signaling Promotes JAK/STAT Activation

Ros generated during apoptosis promotes tolerable levels of JNK in nearby surviving cells. Thus, in addition to apoptotic cells at the wound site, nonapoptotic cells also appear that have nondeleterious levels of JNK and low levels of Ros [22]. Hemocytes stimulated by Ros are also involved in activating JNK in surviving cells adjacent to the wound, since hemocytes release the TNF ligand Eiger that can induce JNK signaling [25]. JNK signaling plays a key role in regulating many biological processes involved in regeneration, including wound healing, compensatory proliferation, apoptosis, and cytoskeletal rearrangement. Inhibition of JNK during disc regeneration impairs wound healing and reduces regenerative proliferation [8, 28, 45, 46]. These regenerative responses depend on the activation of several downstream pathways by JNK.

**Figure 3:**
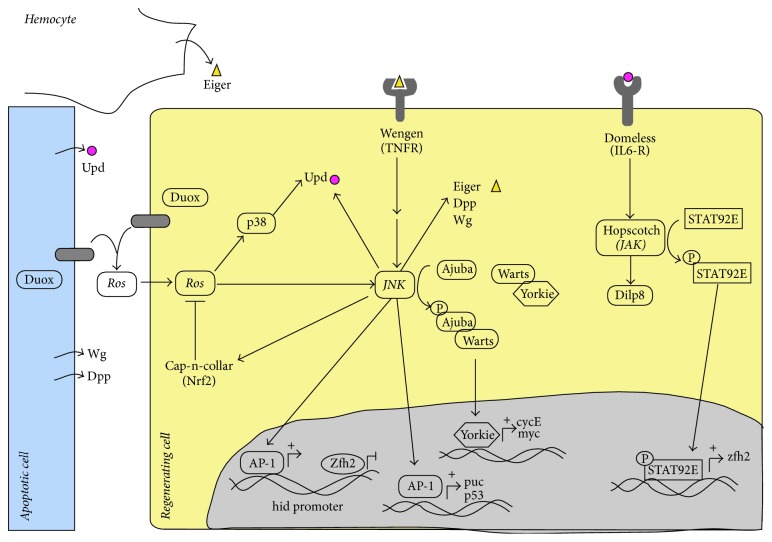
Schematic of regulatory interactions between components of signaling pathways involved in promoting Drosophila wing disc regeneration. See text for details.

**Figure 4:**
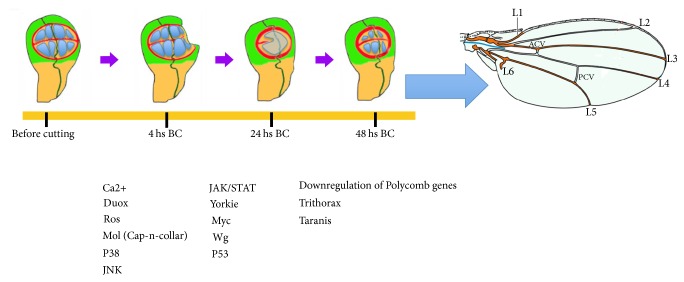
Schematic representation of the different factors and signaling pathways involved in the regulation of the cellular processes that occur during wing disc regeneration; see text for details.

In addition to the activation of JNK signaling, Ros can regulate the P38 stress-activated MAP kinases in surviving cells. The activation of P38 signaling is independent of the JNK pathway [22] and it has been proposed that Ros may promote the P38 pathway through the oxidative modification of intracellular kinases, such as the redox-sensitive activating protein-1 ASK1 [31]. The nondeleterious activation of JNK and P38 MAP kinases by Ros may have multiple effects, among them the induction of cytokine expression [11, 22, 29, 47, 48].* Drosophila* has three leptin-like (IL-6 family) cytokine ligands known as the Unpaired proteins (Upd, Upd2, and Upd3). After physical injury or cell death, the three* upd* genes are upregulated in the wound's edges in a manner dependent on JNK signaling [22, 47, 48]. It is unclear whether these factors are expressed exclusively in dead cells (as we mentioned before), or they are also expressed in surviving cells surrounding the damage region. In this review we have considered that both dead and surviving cells can express these ligands. The Upd ligands bind to the IL-6R type receptor* Domeless (dome)*, which activates the Janus kinase Hopscotch (Hop), and this phosphorylation cascade promotes the translocation of a Stat3-like transcription factor (Stat92E) to the nucleus (Figure 3). All these factors constitute the JAK/STAT signaling pathway, which has important roles in disc development, for instance appendage patterning [49–51] and the control of cell proliferation [49, 52–54]. The elimination of JAK/STAT components during leg disc regeneration impairs local cell proliferation [48]. Similarly, during wing disc regeneration, reduced JAK/STAT activity also partially disrupts adult wing recovery, leading to the generation of much smaller adults wings [22, 47]. As such, it was proposed that JAK/STAT signaling functions downstream of JNK/P38 signaling and that it is necessary to induce compensatory cell proliferation and to form the blastema (Figure 4) [48]. However, instead of promoting compensatory cell proliferation, it has been proposed that JAK/STAT might be necessary to restrain the excessive tissue damage caused by the activation of the JNK pathway, which would facilitate the initiation of compensatory responses [47]. JAK/STAT could act as a suppressor of JNK signaling and this repression could either be mediated by direct transcriptional effects on JNK components or indirectly, by suppressing apoptosis. This mechanism could restrain the nonautonomous activation of JNK and excessive apoptosis [47]. This function of JAK/STAT would be mediated by Zfh1 and Zfh2 (Zinc-finger homeobox) proteins. These ZEB proteins that act as transcriptional repressors [47] have been previously identified to be downstream effectors of JAK/STAT [47, 49, 55]. In the promoter region of* hid* as well as in the promoter of the gene* key*, which encodes for the AP-1 component dFos, appears multiple, highly clustered mammalian ZEB1-binding motifs [47]. Therefore, it has been proposed that Zfh1 and Zfh2 might be restraining JNK activation by repressing* kay*. In addition, ZEB proteins might be also competing with AP-1 for transcriptional repression of* hid*, thereby limiting the apoptosis induced by JNK signaling through* hid* (Figure 3) [47].

In addition to the possible role that JAK/STAT might have in controlling cell proliferation, this pathway also induces a physiological response by activating Drosophila* insulin-like peptide (dilp8)* [47, 48]. This paracrine factor is activated after damage and it regulates the timing of pupariation [56, 57]. It has been reported that Dilp8 regulates both developmental delay and growth coordination between regenerating and undamaged tissue. Dilp8 inhibits the production of the neuropeptide prothoracicotropic hormone (PTTH), causing developmental delay [56, 57]. Moreover Dilp8 activates Nitric oxide synthase (NOS) in the prothoracic gland. NOS limits the growth of undamaged tissues by reducing ecdysone biosynthesis [58, 59]. The function of Dilp8 is mediated by the Orphan leucine-rich G-protein coupled receptor Lgr3. Lgr3 activity is necessary in the Central nervous system (CNS), as well as in the prothoracic gland, for NOS activation following damage [60–62].

As we mentioned the activation of* dilp8* after damage depends on the JAK/STAT pathway [47, 48]; therefore, JAK/STAT signaling might favor regeneration by delaying development [47, 48, 56, 57].

## 5. The Role of Wg and Dpp Signaling in Disc Regeneration

The Wingless family of proteins (Wnt class) are involved in regeneration in different organisms with this capacity [2, 63, 64]. Intriguingly, different responses to* wg* expression have been observed during disc regeneration depending upon the proapoptotic gene employed or the methods of inducing the wound. Thus,* wg* (the Drosophila Wnt1 homologue) is ectopically expressed near the lesion edges before blastema formation in amputated leg and eye imaginal discs [48, 65–67]. In addition,* wg* is upregulated in the wing cells that form the blastema after genetic ablation by expressing* Eiger* or the proapoptotic gene* reaper* [9]. During these processes* wg* is activated by JNK signaling [48]. Using these experimental approaches regenerative proliferation was impaired when* wg* was reduced [9, 48]. Accordingly, it was proposed that* wg* is required for regenerative proliferation (Figure 4). This effect is at least in part due to the down regulation of Notch, which leads to Myc upregulation [9]. It has also been proposed that JAK/STAT signaling cooperates with Wg signaling to induce regenerative cell proliferation [48].

Paradoxically, when apoptosis was induced by overexpressing the proapoptotic gene* head involution defective (hid)* or when a portion of a disc is eliminated* in situ* [12],* wg* expression was not altered during disc regeneration [68]. Moreover, knocking down* wg* did not block discs regeneration after* in situ* amputation or* hid* expression [12, 68]. The basis for these differences in* wg* expression and its requirements are not yet clear. They might in part reflect differences in the efficiency of genetic ablation, or that different methods of inducing a wound elicit different responses in terms of gene expression. Alternatively,* wg* function might be redundant with the activity of other genes of Wnt family present in Drosophila, such as* wnt6*. In fact,* wnt6* and* wg* share the same regenerative enhacer (see below). Therefore, more work is needed to define the role of* wg* signaling in the regenerative response.

The bone morphogenetic protein (BMP) Decapentaplegic (Dpp) activates a signaling pathway that plays an important role in inducing growth and patterning during imaginal disc development [69–72]. Therefore, it was suggested that the Dpp pathway might be redeployed to control regenerative growth. However, the contribution of the Dpp signal to this process remains unclear. Thus, although* dpp* is transcriptionally activated in response to genetic ablation in wing discs [9], this is not the case in amputated wing discs [46]. Moreover, while Dpp is required for the hyperplastic growth caused by “undead” cells, when apoptotic cells are protected with P35 [73, 74], this factor is dispensable for compensatory cell proliferation when P35 is not ectopically expressed in apoptotic cells, even though Dpp is expressed in apoptotic cells [74, 75]. As yet, the basis for these differences remains unclear.

It has been proposed that both* wg* and* dpp* are activated in apoptotic cells and diffuse to surrounding cell to promote proliferation [73–75]. However, as JNK signaling pathway is active, although at low levels, in surviving regenerating cells [22], and* wg* and* Dpp* are targets of JNK signal, we do not exclude the possibility that these factors might be also expressed in some regenerating cells (Figure 3).

## 6. The Hippo Pathway Is Necessary for Regenerative Growth

Hippo signaling is a conserved pathway that regulates growth during development and regeneration, and its deregulation is associated with oncogenesis (reviewed in [76, 77]). This signaling pathway is constituted by a kinase cascade that can be activated by different stimuli. Hippo signaling is mediated by a transcriptional coactivator protein, Yorkie (Yki in* Drosophila*, YAP in vertebrates: reviewed in [77, 78]) (Figure 2). Yki remains inactive when the signaling pathway is active and it is retained in the cytoplasm due to its phosphorylation by the kinase Warts (Wts) [79]. When Wts is inactive, unphosphorylated Yki accumulates in the nucleus [77, 78] and in conjunction with different DNA-binding proteins, it promotes the transcription of downstream genes necessary to promote cell proliferation, such as Cyclin E and cMyc [77, 78] (Figures 3 and 4).

The Hippo pathway plays a key role in inducing regenerative growth after disc damage [80–82]. This pathway can be activated by multiple upstream inputs, including Fat–Dachsous signaling, sense tissue damage, and JNK signaling [82, 83]. JNK signaling can directly promote the activation of Yki by phosphorylating Ajuba family LIM proteins and enhancing their binding to Wts, thereby preventing their activation by Hippo [84]. Interestingly, the ability of JNK to activate YAP is conserved in mammalian cells [83, 84]. Thus, JNK increases Yki activity after wounding, a process essential to induce compensatory cell proliferation and regeneration.

## 7. The Control of Cell Plasticity during Imaginal Disc Regeneration by the Polycomb Group (PcG)

One of the processes associated with organ regeneration is the repatterning of the regenerating tissue, which implies genetic reprogramming of cells in order to switch their fates (Figure 4). After damage, newly formed tissue is derived from surviving cells that lie nearby and some of these cells must change their state of determination to contribute to the lost region.

During disc regeneration several observations indicate that cell fates are respecified and that there is a process of cell reprograming. For example, there is a temporary loss of markers of cell fate commitment after genetic ablation or disc amputation [9, 12, 85]. It has also been reported that after genetic ablation in the wing pouch, the cells of the hinge generate cells that become part of the pouch [68, 86]. Moreover, cell fate changes between compartments have been reported after surgical excision [66] or genetic ablation [87]. Indeed, cells near the anterior/posterior or dorsal/ventral boundary can change their identities and contribute to the compartment on the other side of the boundary [87]. Finally, during regeneration the cells of one disc occasionally acquire the identities of different imaginal discs, switching cell fate and generating disc-inappropriate structures, a process known as transdetermination [88].

The preservation of a specific cell fate or determination state depends on a particular genetic program, which is largely maintained through epigenetic modifications that are established during development. The polycomb group (PcG) proteins function as epigenetic modifiers and they are required to maintain cell fates by controlling the expression of developmental regulators [89]. This group of proteins forms two different types of complexes, Polycomb repressive complex 1 (PRC1) and Polycomb repressive complex 2 (PRC2). PcG can silence large numbers of genes by establishing repressive marks like histone H3 lysine 27 trimethylation (H3K27me3). There is evidence that JNK signaling downregulates PcG genes during regeneration, thereby allowing the transcription of otherwise silenced genes [90]. This process is important for cell reprogramming during regeneration. Inappropriate or excessive downregulation of the PcG by JNK during regeneration may activate genes that induce a genetic program corresponding to a different disc, provoking transdetermination. Indeed, the frequency of transdetermination is enhanced in PcG mutant discs [90]. Interestingly, ectopic activation of* wg* can induce transdetermination, possibly because* wg* might be a direct target of the PcG [90].

The preservation of the anterior/posterior compartment identity during regeneration is mediated by* taranis (tara)*, that is, the homologue in Drosophila of the vertebrate TRIP-Br (Transcriptional Regulators Interacting with plant homeodomain (PHD) zinc fingers and/or Bromodomains) family of proteins. In mutant conditions for* tara, *regenerating wing disc undergoes posterior-to-anterior transformations late in regeneration. These changes are consequence of the misregulation of posterior selector gene* engrailed (en)*. The deregulation of* en* leads to the autoregulatory silencing of the* engrailed* locus, which requires the PRC1. The misregulation and subsequent silencing of* en* are induced by JNK signaling. It has been proposed that Tara stabilizes* engrailed* expression downstream of JNK signaling to maintain the posterior cell fate identity during regeneration [91].

Recently, a defined regulatory element was identified that is responsible for the activation of* wg* expression after damage [92]. Interestingly, this regenerative enhancer (BRV118) regulates the expression of* wg* and* wnt6*. This observation suggests that the function of different members of Wnt family might be involved during regeneration in Drosophila. It has been described that within this enhancer there is a damage-responsive module that remains active throughout the third instar stage and an adjacent silencing element that nucleates increasing levels of epigenetic silencing during development. This latter element can restrict the activity of this enhancer [92]. Therefore, the loss of the regenerative capacity of the discs as development proceeds might be explained by a blockade of the damage-responsive enhancers through the activity of the silencing elements. This mechanism might prevent gene expression in the mature organism without compromising the gene activity regulated by developmental signals [92]. Interestingly, PcG-mediated epigenetic silencing is required to regulate the activity of this enhancer. Hence, the inability of the cells in adult tissue to reactivate programs necessary to promote regenerative growth or cell fate respecification could limit regeneration in adult stages.

## 8. Perspectives

The urodele amphibians have been used extensively as a model system to study regeneration as they present a remarkable regenerative capacity and they can fully regenerate amputated appendages [93]. While the studies carried out on these organisms allowed multiple cellular processes involved in limb regeneration to be identified [93], much less is known about the genetic mechanisms that control them, as amphibians are not the best model organisms for genetic analyses. Moreover, most studies into regenerative biology aimed at developing biomedical applications have been carried out on stem cells cultivated* in vitro*. To better understand the processes that occur during regeneration, these phenomena must be studied* in vivo*, in the context of the complex genetic and cellular interactions that take place. Drosophila is a complex model organism in which the mechanistic details of genetic and cellular processes can be defined. In addition, Drosophila has been extensively used as a model system to carry out unbiased genetic screens to identify genes involved in different cellular processes. These features make Drosophila an excellent model to identify and characterize genes involved in all aspects of regeneration. In fact, different genetic screens and studies of the changes in gene expression during disc regeneration have identified multiple signaling pathways and genes required for different processes associated with regeneration [23, 24, 94].

The conservation between flies and vertebrates of basic signaling pathways and their regulatory elements justifies using Drosophila as a model organism to establish mechanisms and genetic processes that can be translated to vertebrates. Different studies have confirmed that most of the signaling pathways required for disc regeneration are also involved in regeneration in vertebrates; for example, the Hippo pathway appears to play a fundamental role in vertebrate limb regeneration and in skin wound healing [95, 96]. JNK is very important in mammalian liver regeneration and one of its targets, the AP-1 transcription factor subunit c-Jun, is activated during liver regeneration [97–99], the cytokines TNF-*α* and IL-6 also being required during this process [99]. Finally, and as in Drosophila, PcGs are downregulated during murine skin repair, which provokes the derepression of* dmyc* [100]. Moreover, it has been suggested that that loss of polycomb-mediated silencing might contribute to the induction of repair genes in mammals.

In summary, basic regenerative research carried out in Drosophila can provide insights into the genetic and cellular responses involved in mammalian regeneration. This knowledge might serve to develop new therapies in regenerative medicine.
